# Varied Genomic Responses to Maladaptive Gene Flow and Their Evidence

**DOI:** 10.3390/genes9060298

**Published:** 2018-06-12

**Authors:** Marius Roesti

**Affiliations:** Biodiversity Research Centre and Zoology Department, University of British Columbia, Vancouver, BC, V6T 1Z4, Canada; roesti@zoology.ubc.ca

**Keywords:** comparative genomics, divergence with gene flow, genome architecture, genomic clustering, divergence mapping, chromosomal inversion, introgression, quantitative trait locus (QTL) mapping, recombination, threespine stickleback

## Abstract

Adaptation to a local environment often occurs in the face of maladaptive gene flow. In this perspective, I discuss several ideas on how a genome may respond to maladaptive gene flow during adaptation. On the one hand, selection can build clusters of locally adaptive alleles at fortuitously co-localized loci within a genome, thereby facilitating local adaptation with gene flow (‘allele-only clustering’). On the other hand, the selective pressure to link adaptive alleles may drive co-localization of the actual loci relevant for local adaptation within a genome through structural genome changes or an evolving intra-genomic crossover rate (‘locus clustering’). While the expected outcome is, in both cases, a higher frequency of locally adaptive alleles in some genome regions than others, the molecular units evolving in response to gene flow differ (i.e., alleles versus loci). I argue that, although making this distinction is important, we commonly lack the critical empirical evidence to do so. This is mainly because many current approaches are biased towards detecting local adaptation in genome regions with low crossover rates. The importance of low-crossover genome regions for adaptation with gene flow, such as in co-localizing relevant loci within a genome, thus remains unclear. Future empirical investigations should address these questions by making use of comparative genomics, where multiple de novo genome assemblies from species evolved under different degrees of genetic exchange are compared. This research promises to advance our understanding of how a genome adapts to maladaptive gene flow, thereby promoting adaptive divergence and reproductive isolation.

## 1. Introduction

A central interest in evolutionary biology is to understand where adaptation and divergence of populations occurs within a genome. Comparisons of adjacent populations from disparate environments often reveal strong differentiation in some genome regions, particularly in genome regions with a low crossover rate (e.g., [1,2,3,4,5,6,7]). A likely explanation for this bias is that local adaptation in the presence of maladaptive gene flow (from here on simply ‘gene flow’) profits from the non-random genetic association of loci (linkage disequilibrium (LD)) within a genome. In a genome region where crossover happens only rarely, locally adaptive alleles (hereafter simply ‘local alleles’) can be brought into strong LD, allowing them to resist gene flow jointly and thus more efficiently. In contrast, fixation of local alleles is more difficult in a genome region where the crossover rate is high and introgression of foreign alleles relatively frequent [8,9,10,11,12,13,14,15,16,17]. Most local alleles will therefore be swamped by gene flow in high-crossover genome regions [18], whereas in low-crossover genome regions, a higher frequency of local alleles can be maintained, resulting in stronger differentiation between divergent yet connected populations [14,15,16,17].

The purpose of this perspective is to discuss how the above idea can be applied in several ways to explain why local alleles may become more frequent in some genome regions as a consequence of gene flow during adaptation, particularly within genome regions with low crossover rates. I highlight the importance of being explicit about the molecular units, that is to say alleles or loci, evolving in response to gene flow. Furthermore, I consider theory and conduct empirical analyses of threespine stickleback quantitative trait loci (QTL) to argue that deciphering the nature of the adaptive response of a genome to gene flow is often challenging and requires rigorous methodological consideration.

## 2. ‘Allele-Only Clustering’: When Selection and Gene Flow Build Clusters of Alleles, but not of Loci

To begin, let us focus on cases where gene flow influences only the frequency of local alleles across a genome, but not the actual arrangement of the loci that are relevant for optimal adaptation to a local environment (hereafter called the ‘relevant loci’).

If two relevant loci happen to be close by on a chromosome, selection can build up strong LD between the local alleles, allowing them to resist gene flow more efficiently than local alleles at relevant loci located far apart from one another [9,12,14]. However, the degree to which selection can build LD in response to gene flow is not a simple function of the physical proximity of the relevant loci, but it strongly depends on the crossover rate (cM/Mb) [15]. In genome regions with a relatively low crossover rate, LD can become stronger, thus facilitating local adaptation with gene flow [5,8,9,13,14,17,19]. Indeed, empirical evidence highlights that adaptive divergence between connected populations is often stronger in chromosome centers and on larger chromosomes where the crossover rate in eukaryotic genomes is typically low (hereafter referred to as ‘typical low-crossover genome regions’ [6,17,20]). An important insight is that stronger differentiation in low-crossover genome regions does not necessarily indicate that relevant loci are more numerous in these genome regions. For example, simulations have shown that divergence with gene flow can evolve to be stronger in typical low-crossover genome regions even when relevant loci are more frequent in high-crossover genome regions [17].

**Figure 1:**
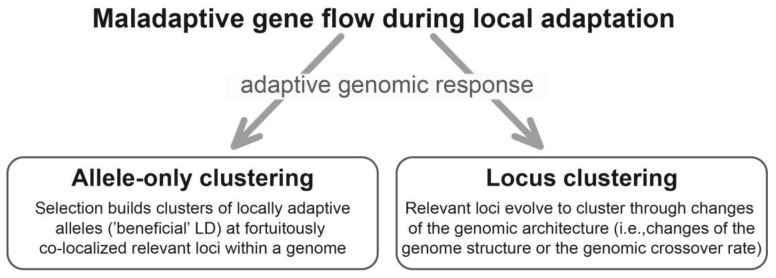
Schematic overview of allele-only clustering and locus clustering. LD: linkage disequilibrium.

From here on, I use the term ‘allele-only clustering’ to refer specifically to cases where local adaptation with gene flow profits from fortuitous co-localization of relevant loci within a genome (Figure 1). This is the case when relevant loci happen to co-localize, such as within a typical low-crossover genome region, for any reason other than having evolved to do so through changes of the genomic architecture in response to gene flow (see following section). Because allele-only clustering means that selection builds genomic clusters of local alleles—but not of relevant loci—in response to gene flow, it should be understood as one possible evolutionary response of a genome to maladaptive gene flow.

## 3. ‘Locus Clustering’: When Selection and Gene Flow Build Clusters of Loci

In theory, selection for LD among relevant loci in response to gene flow can also result in the genomic co-localization of the relevant loci themselves [13,14,19,21]. In what follows, I outline how such ‘locus clustering’ may involve selection-driven changes of the physical (i.e., the arrangement of loci) or functional (i.e., the crossover rate) genome structure, to which I will jointly refer to as adaptive changes of the ‘genomic architecture’ in response to gene flow (Figure 1).

First, selection may act to co-localize relevant loci in response to gene flow through molecular mechanisms, such as genetic deletions or transpositions [22], which can bring loci closer together within a genome. Here, one could speculate that relevant loci may often evolve to co-localize within typical low-crossover genome regions. Support for this idea comes from the theoretical and empirical observation that adaptive divergence appears less constrained by gene flow in typical low-crossover genome regions [6,17,23], indicating the potential benefit for local adaptation if relevant loci were to evolve into these genome regions. Also, simulations by Yeaman (2013) [19] have shown that when given the chance to move position along a chromosome, relevant loci evolve to become physically coupled during local adaptation with gene flow. Arguably, this could be achieved relatively easily through the co-localization of relevant loci within low-crossover genome regions. Finally, convincing empirical support for the idea that low-crossover genome regions may serve as co-localization hotspots for relevant loci comes from sex chromosome evolution; males and females of the same species can be thought of as two genetically connected populations evolving towards different fitness optima because many trait values that are optimal in one sex are suboptimal in the other sex. This conflict is often resolved through the evolution of divergent sex chromosomes between which crossover is largely arrested [24]. The sex chromosome present in only one of the sexes (e.g., the Y in XX/XY systems) then recruits genes with sex-specific functions from autosomes [25]. In short, divergence between males and females evolves through co-localization of genes within a genome region with suppressed crossover between the sexes.

However, selection may create LD not only by moving relevant loci closer together and possibly into genome regions where the crossover rate is low already, but also by acting to reduce the crossover frequency between relevant loci in response to gene flow. A chromosomal inversion, for example, creates two local chromosome variants between which crossover is largely suppressed [12,26,27,28,29,30]. If one inversion variant happens to capture local alleles at multiple relevant loci, these alleles can resist gene flow jointly and thus relatively easily [12,27,28,31,32]. This general idea is thought to explain why inversions are often strongly differentiated between divergent yet connected populations (e.g., [33,34,35,36,37,38,39,40]), and why inversion polymorphisms appear to be more abundant in species that have evolved with as opposed to without gene flow (e.g., fruit flies [31] and passerine birds [41]). Notably, the crossover rate between relevant loci may also directly evolve in the absence of structural genome rearrangements, for instance, via genes [42,43,44] or epigenetic DNA modifications [45,46] that can directly modify the crossover rate within a genome.

Taken together, locus clustering should be understood as selection driving genomic co-localization of relevant loci in response to gene flow through structural genome changes or an evolving intra-genomic crossover rate (Figure 1). Here, selection for LD thus results in clustering of the relevant loci within a genome in response to gene flow, which contrasts with allele-only clustering wherein the molecular units evolving to cluster are alleles only.

## 4. Is a Low Crossover Rate Indeed Advantageous to Adaptation with Gene Flow?

So far, I have pointed out only disadvantages of crossover for local adaptation with gene flow, and reasoned that low rates of crossover in some parts of a genome may be key for both allele-only and locus clustering. This poses the question: Is crossover all that negative when local adaptation occurs in the presence of gene flow?

There are at least two reasons why some crossover between relevant loci should be advantageous even when a population adapts under gene flow. First, crossover allows for the linking of local alleles that are initially on different chromosome copies into the same copy, thus creating LD [47,48,49,50]. So, although crossover can break favorable allelic associations and thereby hinders adaptation in the presence of gene flow, it can also be essential for building these associations in the first place. This positive effect of crossover is thus expected to be relatively small when local alleles are in strong LD within a population already [51,52].

A conceptually related benefit of crossover to a population is that through the breaking and rejoining of different chromosome copies, crossover allows for the purging of deleterious genetic variants from the gene pool [47,48,53,54,55,56]. Such deleterious variants can include unconditionally deleterious mutations, or alleles at ecologically relevant loci that are selected against in the local environment. In fact, the difficulty of purging deleterious variants when the crossover rate is extremely low or absent entirely, as it is the case in the immediate genomic neighborhood of centromeres or on the sex chromosome that is present only in the heterogametic sex, probably explains why these genome regions have evolved to be relatively gene poor [22,57,58]. Across a wide range of organisms, however, typical low-crossover genome regions are not, or are only slightly, reduced in gene content [17,20], implying that crossover is frequent enough in these regions to counteract possible negative fitness effects of linkage. When the crossover rate is reduced in a genome region due to a structural genome rearrangement, it is worth mentioning that crossover suppression is strong only in individuals that are heterozygous for such a structural polymorphism [30] and that the purging of deleterious variants can be ongoing in homozygous individuals.

In short, while reduced crossover between relevant loci can facilitate adaptation in the presence of gene flow, some degree of crossover seems essential. Clearly, further theoretical investigation should be devoted to quantifying this ‘optimal range’ of a low, but not too low, crossover rate for adaptation with gene flow [6,44], an endeavor that is likely to be complex [51].

## 5. On the Evolutionary Origin and Implications of Locus Versus Allele-Only Clustering

Both locus and allele-only clustering should be thought of as adaptive changes of a genome driven by selection favoring LD among relevant loci in response to gene flow (Figure 1). So, is it necessary to distinguish whether local alleles only, or the relevant loci themselves, evolve to cluster within a genome? I argue that these processes likely differ in frequency and relative importance under different evolutionary circumstances.

First, let us reconsider locus clustering involving adaptive changes of the physical genome structure. In general, the chance for a beneficial structural genome change to occur and establish within a population in response to gene flow seems relatively low and to require rather specific biological circumstances [32,59,60]. Also, some genome regions seem more prone to structural changes than others in the first place [61,62], indicating that the rate at which structural clusters of relevant loci can evolve may be low in some genome regions. Furthermore, large inversions linking many local alleles, and therefore bringing the greatest benefit to adaptation with gene flow [12,63,64], are also predicted to have relatively high fitness costs for heterozygous individuals [63,64,65]. The evolution of a structurally adapted genome to a local environment is thus likely to take relatively long (i.e., many thousands to millions of generations [19,25,41]). Once evolved, however, the importance of inversions and other structural genome re-arrangements may be substantial for adaptation with gene flow, perhaps making them particularly persistent and abundant in species with an evolutionary history of sustained gene flow. In contrast, locus clustering involving (epi-)genetic changes of the crossover rate may evolve on much shorter timescales [44,66]. For example, Charlesworth and Charlesworth (1985) [67] successfully selected—in less than 18 generations—for a change in the crossover rate between specific genes in fruit flies, and demonstrated that the evolved crossover change was not due to a structural genome rearrangement.

A higher frequency of local alleles in some genome regions due to allele-only clustering is expected to be relatively common in nature because local adaptation often occurs with some gene flow [68,69] and every eukaryotic genome is likely to exhibit some fortuitous co-localization among the relevant loci, which are commonly numerous [20]. Simulations have shown that strong LD among pre-existing local alleles at fortuitously clustered loci in low-crossover genome regions can build up within a few hundred generations during adaptation with gene flow [17]. However, the efficiency and speed of this process likely depends on pre-existing LD in a population [6,36,70,71]. When considering de novo mutation, a new allele is more likely to establish in a population when it arises linked to other positively selected genomic variation in the presence of gene flow, although the establishment probability of a new local allele will depend mainly on its selective effect size [19,72,73,74,75]. Such a condition may, for instance, be given when a new local allele arises within an inversion that is already segregating between two connected populations [60,72,73].

This last example indicates that locus and allele-only clustering are very challenging to differentiate empirically because it often remains unclear whether a change of the genomic architecture evolved primarily in response to gene flow [60,76]. Inversions are a case in point of this uncertainty because one inversion-variant may become frequent in a population due to positive effects other than that of linking loci [77,78,79], or because of pure chance (drift), assuming that the inversion is not or is only mildly deleterious. Such an inversion could then, secondarily, provide the substrate for new local alleles to establish and rise in frequency at relevant loci fortuitously captured by the inversion [60,72]. Similarly, it is often unclear whether low crossover indeed facilitated the initial build-up of local adaptation in a genome in the face of gene flow. This is because local adaptation may have occurred mainly during periods where a population was not genetically connected to a foreign population. In this case, low crossover would mainly facilitate the *maintenance* of local alleles in the presence of gene flow [17,72,80]. Considering the above points collectively, inferring the precise evolutionary history of clustered adaptive alleles within a genome appears challenging empirically. Before addressing this problem, however, we first need to ask: Can the clustering of *adaptive* alleles (due to allele-only or locus clustering) be reliably detected within a genome at all?

## 6. The Use of Divergence Mapping to Quantify Adaptive Clustering within a Genome

Putative empirical evidence for adaptive genomic clusters of local alleles, and potentially of relevant loci, could come from divergence mapping, where genome-wide polymorphisms are screened for regions with accentuated differentiation between divergent populations. These ‘outlier’ regions are then thought to pinpoint genome regions targeted by natural selection during local adaptation [81,82]. However, there are good reasons to believe that localizing and quantifying selected sites reliably across a genome through differentiation outliers is problematic. These concerns have been discussed extensively before (see e.g., [17,20,80,83,84]), so I will here outline them only briefly and specifically with regard to testing for genomic clustering of local alleles and/or relevant loci in response to gene flow.

The main concern with divergence mapping for quantifying and localizing local adaptation across a genome is related to intra-genomic crossover rate variation. In a low-crossover genome region where LD is generally strong, not only a locus targeted directly by divergent selection will show increased differentiation, but also an extensive part of its associated (neutral) genomic neighborhood [85]. In contrast, the signature left around a selected locus will be narrower in a genome region with a higher crossover rate (assuming that the strength of selection on the locus is the same). Importantly, the degree to which crossover affects how far the effect of selection extends into the genomic neighborhood of a selected locus is of particular relevance in local adaptation with gene flow because it determines how easily gene flow homogenizes the genome between populations around a locus under divergent selection.

This general insight has several implications for the use of divergence mapping in detecting adaptive alleles and relevant loci within a genome. First, relatively narrow selection signatures in high-crossover genome regions—and hence the underlying relevant loci—are likely to be missed. Second, it generally remains unclear how many of the strongly differentiated alleles in a genomic outlier region are actually adaptive, and hence, how many loci are under local selection in this genome region. Third, the impact of non-local selection (e.g., background selection or selection against ecologically-unrelated genetic incompatibilities) will also be stronger in low-crossover genome regions [86,87,88], thus leading to a higher rate of false positives in genome regions with low crossover rates [80,89,90].

Fortunately, there are strategies for dealing with these problems. First, whole genome instead of reduced-representation genome sequencing should be applied so that relevant loci are sequenced directly and can be localized more precisely. However, if whole genome sequences are analyzed by coarsely integrating markers along a genome (i.e., data smoothing) without accounting for genomic heterogeneity in LD due to crossover rate variation, the detection of differentiation outliers will remain biased towards low-crossover genome regions [20,84]. Second, replicate population comparisons should be analytically integrated because the repeated detection of an outlier genome region in independent and ecologically similar comparisons allows for a more reliable detection of loci implicated in *ecological* divergence [69]. However, low crossover rates can result in shared outlier genome regions even when the underlying selection has not targeted the exact same loci [17], thus increasing the rate of false ecological selection outliers in low-crossover genome regions.

Considering these points in concert, larger outlier regions or a greater number of high-differentiation polymorphisms in genome regions with low crossover rates do not inform conclusively on the question of whether local alleles, or even relevant loci, are enriched within these genome regions. Yet, a study comparing the strength of a negative association between crossover rate and differentiation in young populations diverged with versus without gene flow may provide a good indication of whether local adaptation has profited from LD among relevant loci (see [6,17] for more details). This is because such a study tests explicitly whether stronger LD (due to low crossover) has facilitated adaptive divergence in the face of gene flow by focusing on differential gene flow regimes, as well as on populations that have diverged recently. The latter aspect is important because the influence of non-ecological processes potentially mimicking adaptive genomic clustering can be reduced when focusing on recent population divergence [88]. Although such a study may yield evidence that stronger LD in low-crossover genome regions has facilitated local adaptation with gene flow, it is nonetheless unable to distinguish to what degree LD involves locus versus allele-only clustering.

## 7. Does QTL Mapping Allow Testing for *Adaptive* Genomic Clustering and Distinguishing Locus from Allele-Only Clustering?

At first, QTL mapping appears as a promising alternative to divergence mapping to test for adaptive genomic clustering, and to potentially distinguish locus from allele-only clustering, because this method does not rely on indirect genomic selection signatures in the search for relevant loci. Rather, loci controlling quantitative traits can be directly mapped within a genome by a statistical association between genetic variation and phenotypic variation, commonly within an artificially crossed F2 hybrid population. If this mapping population stems from a cross between parents from two divergent populations with a history of gene flow, genomic clustering of mapped QTL for traits involved in local adaptation may thus be assumed to indicate locus clustering.

To evaluate this idea, I leveraged a collection of several hundred QTL compiled by Peichel and Marques (2017) [91] from studies that mapped ecologically-important trait variation in threespine stickleback (*Gasterosteus aculeatus*; Methods S1 provide full details). Notably, marine stickleback have repeatedly adapted to various freshwater habitats under extensive gene flow since the last glacial retreat about 10,000 years ago, a process that is likely to have occurred throughout previous interglacial periods as well [71,92,93,94,95,96,97,98]. Indeed, this makes the threespine stickleback a promising candidate to have evolved adaptive (locus) clustering.

**Figure 2:**
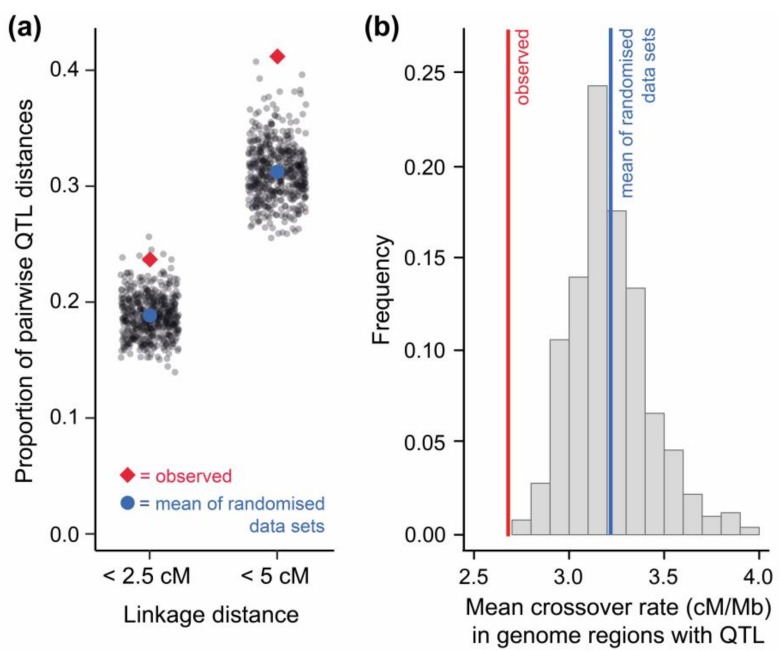
Genomic clustering of stickleback quantitative trait loci (QTL), and the relevance of low crossover genome regions therein. (**a**) The proportion of pairwise QTL distances shorter than 2.5 and 5 cM is higher among real QTL than among an identical number of QTL placed randomly within the stickleback genome (total number of such ‘randomized data sets’ = 500; gray dots indicate the extent of clustering in a single randomized data set). To account for the possibility that one locus may affect several traits (pleiotropy), only unique QTL positions (*N* = 336) were considered for this analysis (Figure S1 presents an analysis including redundant QTL positions). (**b**) The average crossover rate in the genome regions harboring the real QTL is lower than in genome regions of QTL placed randomly within the stickleback genome (histogram indicates the frequency of mean crossover rates in 500 randomized data sets). Notably, heterogeneity in gene density across the stickleback genome was considered when generating the randomized QTL data sets for both (**a**) and (**b**) (see Methods S1).

I first tested for QTL clustering and found that QTL were, on average, closer together in linkage distance (measured in centimorgans (cM)) than expected by chance (Figure 2a, Methods S1). This indicates that ecologically-relevant QTL do not only occur non-randomly within the stickleback genome with respect to their physical position [91], but also with respect to the frequency of crossovers occurring between them (see also [99]). Similar evidence comes from QTL of traits distinguishing hybridizing sister species of European campion flowers [100], ecotypes of Australian groundsel flowers [101], divergent life history types of a North American trout species [102], and salmonid fish in general [103]. Next, I used genome-wide crossover rate data available for threespine stickleback [23] to associate each QTL with a crossover estimate from its respective genome region. This allowed me to test whether QLT clustering involves co-localization of QTL within low-crossover genome regions. Mapped stickleback QTL were clearly over-represented in low-crossover genome regions (Figure 2b), and tended to be enriched on chromosomes with a low overall crossover rate (Figure S2).

**Figure 3:**
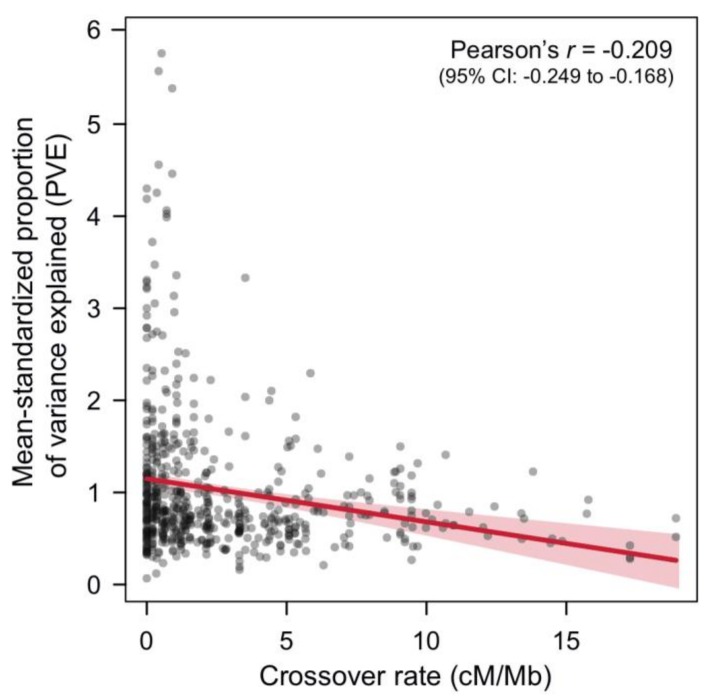
An analysis in threespine stickleback suggests a widespread detection bias of QTL towards genome regions with low crossover rates. Shown is the association between the proportion of phenotypic variance explained (PVE; standardized among studies by dividing the PVE of each QTL by the study’s mean PVE) and the crossover rate for stickleback QTL implicated in adaptive trait variation (see also Table S1). Each gray dot represents one QTL (*N* = 602). In the top right corner, Pearson’s *r* correlation between standardized PVE and crossover rate along with its 95% bootstrap confidence interval (CI) are given. The linear regression fit including the 95% confidence bands are visualized in red.

Indeed, it is tempting to take these findings from stickleback as evidence for an evolved co-localization of QTL in low-crossover genome regions. However, there is a problem with this interpretation because the detection of QTL is generally biased towards genome regions with low crossover rates (see [104] for full details). Genomic blocks in LD are larger in low-crossover genome regions within a mapping population and are therefore likely to span several individual QTL, increasing their chance of detection by mimicking a single QTL of larger effect [104]. In contrast, QTL are often missed in high-crossover genome regions because their individual effects are less likely to be detected as a single combined-effect QTL. A key prediction of this suggested detection bias in QTL mapping is therefore that the effect size of *individual* QTL on the total trait variation will be overestimated in low-crossover genome regions [104]. If so, the proportion of (trait) variance explained (PVE) of *detected* QTL should be higher in genome regions with lower crossover rates. I evaluated this possibility and found a clear negative association between PVE and crossover rate in the stickleback QTL data set (Figure 3; this result was robust to different data filtering strategies, see Table S1). Admittedly, this finding can be interpreted in different ways. On the one hand, it probably implies that the detected large-effect QTL in low-crossover genome regions fractionate into multiple smaller-effect QTL [105], thus suggesting that QTL are indeed more frequent in these genome regions.

On the other hand, it is unclear how many true QTL are currently missed in genome regions with higher crossover rates. This casts doubt on the general reliability of QTL quantification relative to crossover rate variation across a genome [104,106]. Finally, this finding could as well represent a true biological pattern, suggesting that there are more QTL with a larger-than-average individual effect size in low-crossover genome regions. However, there is no obvious reason why especially large-effect QTL should evolve to co-localize in genome regions where the crossover rate is low, nor particularly in response to gene flow, because local alleles at large-effect loci should be able to resist gene flow without being linked to other relevant loci. In contrast, low-crossover genome regions might heuristically be expected to instead harbor clusters of mainly small-effect relevant loci, as these may confer a proportionally greater fitness benefit from being linked to other relevant loci [14,59,107].

**Figure 4:**
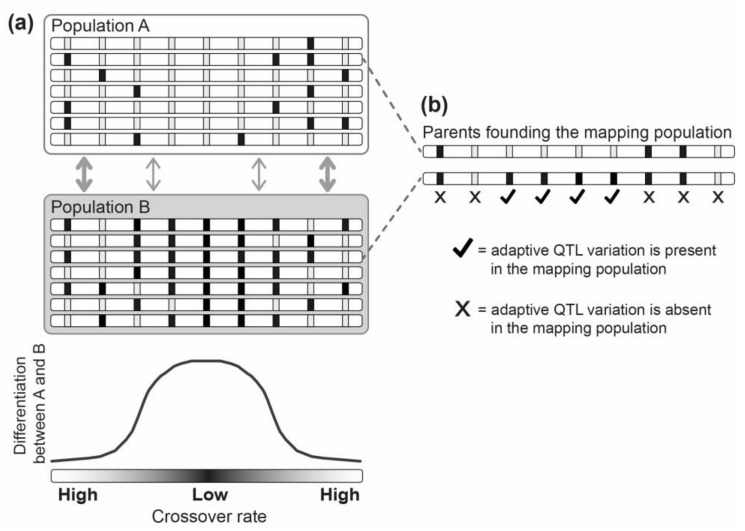
Although called ‘quantitative trait *locus*’ (QTL) mapping, this method cannot distinguish between locus and allele-only clustering. (**a**) Shown are two exemplary populations adapted to different local environments under genetic exchange. Each population is represented by seven individuals, and each individual is illustrated as a single haploid chromosome. The boxes within the chromosomes indicate positions of loci influencing locally adaptive trait variation (relevant loci), while the different gray tones of the boxes represent the alternative local alleles. We assume that all relevant loci have the same selective effect size and that they have not evolved to cluster within the genome in response to gene flow (such a priori knowledge on the distribution of relevant loci is, of course, absent in a real study). In the illustrated case, local adaptation with gene flow is therefore facilitated only by fortuitous co-localization of relevant loci within the low-crossover chromosome center (the thickness of the gray arrows indicates the degree of effective gene flow between the populations). The result is stronger differentiation in the chromosome center because of allele-only clustering. (**b**) When the parental individuals used for a QTL cross are sampled from the two depicted populations, genetic variation at relevant QTL, and therefore QTL detection in general, will be biased towards the high-differentiation chromosome region. Overall, this highlights that interpretations of QTL mapping results should consider the degree to which the parental mapping populations are differentiated and connected by gene flow (see also [107]).

Irrespective of which of these explanations explains the detected pattern best, let us reconsider whether QTL mapping is, in general, a suitable approach to distinguish between locus and allele-only clustering. Here, it is noteworthy that a typical QTL mapping study involves crossing two parental individuals to obtain a hybrid population for mapping. This constrains the detection of QTL to the variation initially present in two individuals [105,108]. Something rarely considered is that the detectable variation at QTL is not only limited overall, but is also biased towards genome regions differentiating the parental populations most strongly. To illustrate this point, let us assume that the two parents used to found a QTL mapping population are chosen from two divergent natural populations exhibiting stronger differentiation in low-crossover genome regions due to allele-only clustering, but not due to locus clustering (see Figure 4a). In such a case, the sampled genetic variation at QTL, and hence the detection of QTL influencing locally adaptive trait variation in general, will be biased towards low-crossover genome regions (Figure 4b). QTL mapping therefore cannot distinguish locus from allele-only clustering, although systematically stronger clustering of detected QTL involving parental individuals from populations that have adapted with versus without gene flow could be used for testing whether adaptive alleles have in general—that is, due to locus and/or allele-only clustering—evolved to cluster within a genome in response to gene flow.

Together, these insights emphasize the general notion set forth by Noor et al. (2001) [104], which is that “QTL maps should be treated as hypotheses to be tested by additional genetic methods”. However, *any* method that is detection-biased by crossover rate variation is not suitable to critically assess the relevance of crossover variation in adaptive evolution, nor will integrating multiple such methods fully remedy this problem. This is also why caution is warranted if the co-localization of QTL (detected through QTL or association mapping) with population differentiation outliers (detected through divergence mapping) is used to infer selection-relevant phenotypes or the degree to which the same genes are re-used in parallel phenotypic evolution in wild populations.

## 8. Summary and Comparative Genomics for a Promising Outlook

In summary, allele-only clustering—that is, the clustering of local alleles at fortuitously co-localized loci as a consequence of local adaptation in the presence of maladaptive gene flow—is expected to be common in nature. This is supported by the view that local adaptation often occurs with maladaptive gene flow and involves many genome-wide loci, which likely vary in their degree of linkage for reasons other than having evolved to do so through architectural genome changes in response to gene flow. In comparison, empirical evidence for the theoretical idea that the actual loci relevant for local adaptation evolve to co-localize within a genome in response to gene flow—potentially within genome regions with low crossover rates—is sparse (but see e.g., [31,41]). While allele-only and locus clustering are not, of course, mutually exclusive processes within a genome, it is an important insight that many of our currently applied methods and study designs are unable to distinguish between these two alternatives.

Future empirical effort should be explicitly directed towards addressing how often stronger local adaptation in certain genome regions is the consequence of fortuitous, or rather of adaptively evolved genomic co–localization of relevant loci. In particular, we should address the role of typical (i.e., ‘meiotically constrained’ [20]) and ‘evolvable’ crossover rate variation within a genome in co-localizing relevant loci in response to gene flow. In this endeavor, divergence and QTL mapping studies should not be blind to their detection bias towards low-crossover genome regions and go beyond simple genome-wide correlations between divergence outliers or QTLs and crossover rate variation as the basis for a biological argument. Also, when a change of the genomic architecture (such as a chromosomal inversion) is detected reliably, it is often safest to first report it as a pattern, agnostically with respect to the underlying evolutionary process. This is because it often remains unclear whether such a polymorphism has evolved as a primary response to gene flow.

**Figure 5:**
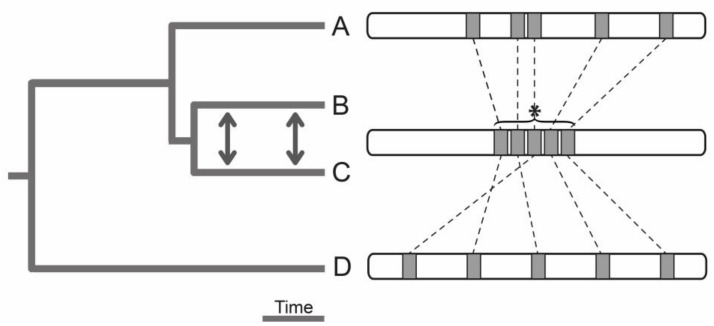
Conceptual outline for the inference of a genome structure evolved in response to gene flow. Compared are de novo genome assemblies from multiple populations or related species (hereafter simply ‘species’) evolved under various degrees of genetic exchange. If a genome region important for local adaptation with gene flow is known a priori (here identified between species B and C, and denoted by an asterisk), the degree of genomic clustering of homologous sequences in a related species that evolved without gene flow (species A) should be reduced to be indicative of an adaptive response of the genome structure to gene flow (see also [112]). Such a test should account for the degree of ‘neutral’ overall structural change of a genome and should profit from integrating distantly related species (species D) because the origin of adaptive structural genome variation can be old and pre-date recent species splits [71]. Without a priori knowledge of genome regions important for local adaptation with gene flow, an evolved genome structure in response to gene flow may be detectable by more abundant structural genome differences between species evolved with versus without gene flow. Finally, if genome regions with lower crossover rates are indeed co-localization hotspots of relevant loci, the ratio of total structural change in low to high crossover genome regions should be higher between species evolved with versus without gene flow.

Luckily, there is hope for tackling these challenges through recent developments of powerful sequencing technologies and computational algorithms. The comparison of de novo genome assemblies from multiple related species evolved under different degrees of gene flow is a particularly promising research program for detecting changes of the genomic architecture and to make inferences about their (adaptive) evolution. A possible conceptual study design for detecting structural genome changes in response to gene flow is illustrated in Figure 5. The question of whether the crossover rate evolves within a genome in response to gene flow could be addressed by comparing crossover rates genome-wide between species that have evolved under different degrees of gene flow. A good indication for an adaptive change would be systematically reduced crossover in a genome region in species that have evolved with versus without gene flow, and evidence for this genome region to be implicated in local adaptation. Overall, these considerations highlight that understanding the role of gene flow in adaptive evolution requires studies that make gene flow variation an integral focus of their design.

The basic theoretical idea of relevant loci evolving to co-localize in response to gene flow should be extended with theory addressing whether locus clustering is also likely when local adaptation results from selection on many genome-wide loci with fluctuating [109] or transient [110] and potentially non-additive contributions, as well as when environmental conditions change frequently. The need for this is emphasized by the recent finding that even adaptation to a stable (artificial) environment is likely to be a highly dynamic process in which the loci responding to selection can change frequently [111]. Ultimately, this knowledge will be critical to our understanding of the impact of gene flow on adaptive evolution at the genomic level, and the importance of architectural genome changes in local adaptation and speciation.

## Supplementary Materials

The following are available online at http://www.mdpi.com/2073-4425/9/6/298/s1, Methods S1; Figure S1: Genomic clustering of stickleback QTL based on all QTL, Figure S2: Stickleback chromosomes with a lower average crossover rate tend to have a greater number of mapped QTL, Table S1: Influence of data filtering and PVE-standardization on the correlation between crossover rate and PVE for stickleback QTL, Supplementary references.
