# Supplementary material for: Varied Genomic Responses to Maladaptive Gene Flow and Their Evidence
Source: Genes (Basel). 2018 Jun 12;9(6):298. doi: 10.3390/genes9060298 (PMC6027369; doi:10.3390/genes9060298)
Supplement: Supplementary file 1 [file genes-09-00298-s001.pdf]

SUPPLEMENTARY MATERIALS

Varied genomic responses to maladaptive gene flow and their evidence

Marius Roesti

Department of Zoology, University of British Columbia, 6270 University Boulevard, Vancouver, BC V6T1Z4, Canada

Correspondence: roesti@zoology.ubc.ca

Content:

Methods S1
Figures S1 & S2
Table S1
Supplementary References

Methods S1

Clustering analyses of threespine stickleback QTL: sources of data and filtering

I used a QTL data set from threespine stickleback *(Gasterosteus aculeatus)* compiled previously by Peichel & Marques (2017) [1] from over twenty mapping studies. I used this data set to test for (i) QTL clustering, (ii) the relevance of low-crossover genome regions in QTL clustering, and (iii) a potential detection bias of QTL towards low-crossover genome regions. Detailed information on the QTL data set can be taken from Peichel & Marques (2017) [1].

The raw QTL data were filtered for QTL from threespine stickleback (i.e., QTL from ninespine stickleback were excluded) and from QTL mapping studies (i.e., QTL obtained trough association mapping were excluded) only. If the same phenotypic trait was mapped multiple times, QTL from only one randomly chosen mapping study were kept for further analysis. All QTL from the 'body shape' trait category were excluded from further analysis. The reason for this was that QTL from this trait category (see ref [1]) appeared highly redundant within and among mapping studies because several studies used individual landmark variation from geometric morphometrics for mapping (but see Table 1 for an analysis including the 'body shape' category, which supported the same conclusions). After these filtering steps, the final data set for analysis consisted of 621 QTL.

Notably, the physical QTL positions were in accordance with the improved stickleback reference genome assembly provided by Roesti et al. (2013) [2]. This allowed inferring the genetic position (cM) of each QTL based on its physical position (Mb), and to obtain an estimate for the crossover rate (cM/Mb) in the genome region around each QTL using linkage map and crossover rate information from Roesti et al. 2013 [2]. Because crossover rate estimates were not available along the entire stickleback chromosomes, only 602 out of the 621 QTL could be associated with a crossover rate estimate.

Assessing the degree of genomic QTL clustering

I assessed the degree of genomic QTL clustering by calculating all pairwise QTL linkage distances (i.e., absolute difference in cM between pairs of QTL) within stickleback chromosomes. These distances were then pooled across all chromosomes. The proportion of short linkage distances was used as a measure for the extent of QTL clustering (i.e., the frequency of pairwise distances < 2.5 and < 5 cM). A higher frequency of short linkage distances thus indicates stronger QTL clustering.

Notably, the analysis presented in the main paper was based on unique QTL positions only. This means that when multiple traits (which may or may not be morphologically related) mapped to the exact same QTL position, this position was retained only once for calculating the pairwise linkage distances (number of total unique QTL positions = 336). This strategy was taken to account for possible pleiotropic effects, that is, when the same locus influences several traits. However, it is also possible that QTLs for multiple traits map to the same genome position although the causal loci are not the same, but that the loci are simply very close-by and thus indistinguishable with QTL mapping. The results presented in the main paper (Fig. 2a) can therefore be thought to provide a conservative estimate of the

extent of QTL clustering in the stickleback genome, and QTL clustering was stronger when the full QTL data set was considered for analysis (i.e., all 621 QTL; see Fig. S1).

The extent of QTL clustering in the real data set was compared to the extent of clustering of QTL placed randomly within the stickleback genome, while taking intra-genomic variation in locus (gene) density into account. To create such a 'randomised data set', I first retrieved the start and end position of all unique genes from the ENSEMBL Genome Browser (BioMart) and projected them onto the Roesti et al. (2013) [2] stickleback reference genome using customized *R* scripts [3]. I then calculated the physical midpoint position of each gene, and randomly sampled a number identical to the number of real QTL of these genome-wide gene midpoints. This generated one randomised QTL data set, which was from there on treated like the real QTL data set to assess genomic clustering (see above). In total, 500 randomised QTL data sets were generated and analysed.

Are QTL more often located in low-crossover genome regions than expected by chance?
I averaged the crossover rate estimates across the genome regions with real QTL, and across the genome regions harboring 'random QTL' within each randomized data set. The frequency distribution of the average crossover rates across the 500 randomized data sets was visualized with a histogram.

I further tested for an enrichment of mapped QTL on chromosomes with a generally lower average crossover rate. To do so, I first calculated the average crossover rate (i.e., maximal genetic length [cM] divided by maximal physical length [Mb]) and the number of mapped QTL divided by the number of total unique genes for each chromosome. I then used Pearsons's *r* to quantify the correlation between these two variables across all chromosomes. Statistical significance (the 95% bootstrap confidence interval and the *P*-value) was assessed through resampling the data randomly 10,000 times [4]. I used the *visreg R* package [3] to visualize the linear relationship between the two variables across all chromosomes.

Testing for a possible detection-bias in QTL mapping by correlating PVE with crossover rate
I evaluated whether QTL mapping is likely to be detection-biased towards low-crossover genome regions by testing for a negative association between PVE (proportion of variance explained) and crossover rate across the QTL. Because PVE estimation for a QTL can be influenced by the design of a study, such as by the number of hybrid individuals used for QTL mapping [5], I standardized PVE across studies by dividing each raw PVE estimate by the mean PVE of the respective study. However, analyzing non-standardized PVE values yielded similar results leading to identical conclusions (Table S1).

I tested for an association between crossover rate and PVE across all QTL using Pearson's *r,* and calculated the respective 95% bootstrap confidence interval (and resampling *P*-value) through resampling the data randomly 10,000 times [4]. Because an association between crossover rate and PVE could be strongly driven by the relatively few QTL with an exceptionally high PVE, I also filtered the QTL data set by excluding the 10% of QTL with the highest raw PVE value and re-assessed the correlation between crossover rate and PVE across the remaining QTL. This analysis supported the same overall conclusions (Table S1.

The linear relationship (regression) between crossover rate and PVE including the 95% confidence interval bands were visualized using the *visreg R* package [3]. All data analysis and graphing for this paper was performed in *R* [3].

Figure S1

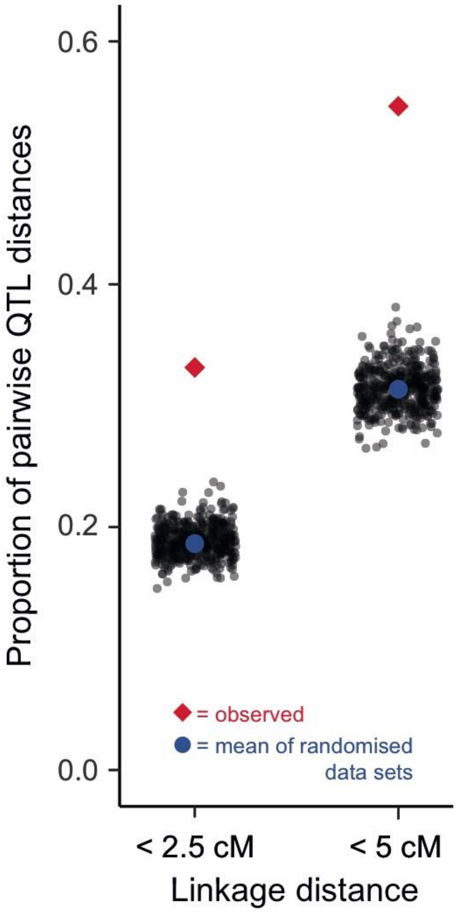

Fig. S1. Genomic clustering of stickleback QTL based on all QTL (i.e., including 'redundant' QTL positions; N = 621). The proportion of pairwise QTL distances shorter than 2.5 and 5 cM is higher in the real QTL data set than in 500 simulated data sets generated by placing an identical number of QTL randomly within the stickleback genome (gray dots indicate single simulation replicates). Plotting conventions follow the ones in Figure 2a.

Figure S2

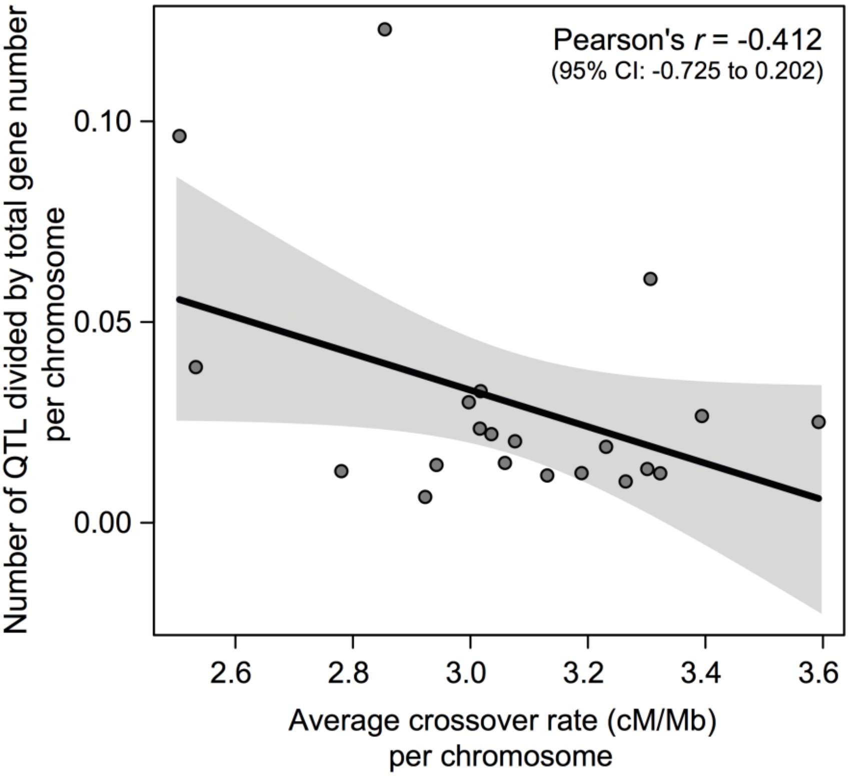

Fig. S2. In stickleback, chromosomes with a lower average crossover rate tend to have a greater number of mapped QTL. Shown is the average crossover rate (cM/Mb) *versus* the number of mapped QTL divided by the number of unique genes per chromosome (gray dots). The black line depicts the linear regression including its 95% confidence bands in gray shading. Notably, the power of this analysis was limited by the number of chromosomes in the stickleback genome (N = 21), which may partly explain why statistical significance is absent for the negative association between overall crossover rate and mapped QTL across chromosomes (resampling P = 0.068).

Table S1. Influence of data filtering and PVE-standardization on the correlation between crossover rate and PVE for threespine stickleback QTL.

**Data filtering**	**Was PVE standardized across studies?**	**Number of QTL**	**Pearson's *r***	**95% confidence interval**	**Resampling *P*-value**
The 'body shape' QTL category was removed	No	602	-0.141	-0.191 to -0.080	0.0010
The 'body shape' QTL category was removed	Yes, by dividing each raw PVE estimate by the mean PVE of the respective study	602	-0.209	-0.249 to -0.168	0.0001
The 'body shape' QTL category was removed; QTL with the highest 10% raw PVE estimates were removed	Yes, by dividing each raw PVE estimate by the mean PVE of the respective study	542	-0.144	-0.214 to -0.070	0.0011
The 'body shape' QTL category was removed; unique QTL positions were considered only	Yes, by dividing each raw PVE estimate by the mean PVE of the respective study	329	-0.149	-0.206 to -0.087	0.0091
Unique QTL positions were considered only (incl. the 'body shape' QTL category)	Yes, by dividing each raw PVE estimate by the mean PVE of the respective study	441	-0.134	-0.187 to -0.075	0.0069

Supplementary References

1. Peichel, C.L.; Marques, D.A. The genetic and molecular architecture of phenotypic diversity in sticklebacks. *Philosophical Transactions of the Royal Society B: Biological Sciences* 2017, *372*.
2. Roesti, M.; Moser, D.; Berner, D. Recombination in the threespine stickleback genome–patterns and consequences. *Molecular Ecology* 2013, *22*, 3014-3027.
3. R Development Core Team. (2017) R: A language and environment for statistical computing. *R Foundation for Statistical Computing: Vienna, Austria.*
4. Manly, B.F.J. (1997). Randomization, bootstrap and Monte Carlo methods in biology. *Chapman & Hall: New York, USA.*
5. Beavis, W.D. (1994) The power and deceit of QTL experiments: Lessons from comparative QTL studies. *Proceedings of the Forty-ninth Annual Corn and Sorghum Industry Research Conference ASTA, Washington,* 252-268.
